# Study on the precursory characteristics and influencing factors of rockburst in the bifurcation area of coal seam

**DOI:** 10.1371/journal.pone.0306811

**Published:** 2024-08-23

**Authors:** Heng Zhang, Caijun Shao, Guofeng Chen, Jincheng Zhou, Wenhao Cao, Xianjun Ji

**Affiliations:** 1 School of Civil Engineering, Nanyang Institute of Technology, Nanyang, Henan, PR China; 2 Henan International Joint Laboratory of Dynamics of Impact and Disaster of Engineering Structures, Nanyang Institute of Technology, Nanyang, Henan, PR China; 3 College of Civil Engineering, Shaoxing University, Shaoxing, Zhejiang, PR China; Amirkabir University of Technology (Tehran Polytechnic), ISLAMIC REPUBLIC OF IRAN

## Abstract

To explore the precursory characteristics and influencing factors of rockburst in the bifurcation area of coal seam, the evolution and expansion of fracture and the energy accumulation and dissipation characteristics of coal-rock parting-coal structure (CRCS) during failure and instability process are explored from a micro-scopic perspective, and the influence of coal and rock parting parameters on the instability is studied. The following four points are addressed: (1) Compared with the single coal structure or the coal- rock combined structure, the CRCS can more directly reflect the geological structure characteristics of the coal seam in the bifurcated area; (2) The failure and instability process of CRCS includes two types of instability: slip and fracture. The slip instability is characterized by low strength and high energy release, which is very difficult to predict. (3) Before the failure of CRCS, there are several precursor signal characteristics, such as the shortened development time of the "stable—fracture—stable" cycle, abnormal slip dislocation of the contact surface, and rapid accumulation of rock fracture energy. (4) The inclination angle of the contact surface affects the instability form, the strength of the rock parting affects the instability state, and the thickness of the rock parting affects the impact tendency. The research results have important theoretical significance for preventing rockburst caused by failure and instability in bifurcated area of coal seam.

## 1 Introduction

Rockburst of structural instability often occur in geological structure with complex coal seam structure [1, 2]. Coal seam with bifurcation is an important geological factor that induces rockburst of structural instability [3, 4]. In recent years, rockburst accidents have occurred frequently in the bifurcation area of coal seam, resulting in a large number of casualties and property losses. Studying the instability forms and influencing factors and exploring the precursory characteristics is highly important in the bifurcation area of coal seam.

Compared with single coal seam structure, studies on the instability of combined coal-rock structure are more in line with field reality [5–7]. In recent years, many scholars have carried out laboratory experiments and field tests on the failure and instability of coal-rock combined structure. Gong et al. [8] used the Split-Hopkinson pressure bar device to conduct dynamic compression experiments on combined coal and rock and studied the stress-strain curve, dynamic peak stress and strain, elastic modulus and the law of energy distribution of coal and rock combination under different loading rates. Wang et al. [9] studied strain softening damage model of coal-rock combination and solution of parameters and explored the influence of model parameters on deformation and failure. These studies have enhanced our knowledge on the fracture and instability of coal-rock combined structure. The presence of rock mass changed the physical and mechanical properties of the original coal seam and enhanced the impact tendency of coal. The mechanical properties included uniaxial compressive strength, elastic energy index, impact energy index and other impact tendency indicators. Jiang et al. [10] explored slip friction experiment on sandstone-coal combined samples under different axial loads via double-sided shear experiment and determined the stick-slip instability characteristics of coal-rock shear slip. Liu et al. [3] explored the instability of coal seam with rock parting by combining field investigation and experimental test and reported that rock parting exhibit stick-slip instability. Unfortunately, this study only discussed the instability process of laminar splices. The above research has enhanced our understanding of the failure and instability process of coal-rock combined structure and has had a far-reaching influence on the study of the structural instability characteristics of rockburst. However, these studies only considered the instability characteristics of combined structure from the perspective of fracture or slip, which is obviously inaccurate [11–14]. Research has focused mainly on two-body "roof-coal seam" or three-body "roof-coal seam-floor" combined structure without considering the impact of rock parting on coal seam structure [15–18]. Changes in the coal seam structure and occurrence state will obviously cause change in its instability characteristics. On the one hand, the existence of rock parting can change the physical and mechanical properties of the original coal seam. These properties include the rockburst tendency indices, such as uniaxial compressive strength, elastic energy index and rockburst energy index. The existence of rock parting enhances the rockburst tendency of coal. On the other hand, abnormal change in coal and rock parting structures easily form stress concentration area, and it is easy to induce rockburst of structural instability when mining activities are carried out in high stress concentration area.

Compared with laboratory experiment and field test, numerical analysis is widely accepted by scholars for its powerful computing power and repeatability [19–23]. Liu et al. [24] studied the mechanical properties and acoustic emission (AE) characteristics of coal- rock combined mass through particle flow code (PFC) in a micro-scopic way and explored the mechanism of crack initiation, propagation and evolution. Cao et al. [25] studied the influence of boundary surface on the mechanical behavior of coal- rock combined mass by using RFPA numerical experiment. They noted that with increasing of contact angle in the combined sample, the instability form changes from coal or rock mass fracture instability to contact surface slip failure. M. Nicksiar and C. D. Martin [26] used the Voronoi method in the universal distinct element code (UDEC) to simulate the formation process of initial fracture in rock and analyzed the effects of block size, distribution characteristics and heterogeneity on crack propagation. He et al. [27] used UDEC to study the dynamic process of roadway rockburst induced by mine earthquake, analyzed the instability mechanism of roadway rockburst under the influence of dynamic road, and identified the main factors affecting roadway instability. The research results were basically consistent with field instability. The application of numerical technology is very beneficial for exploring the instability characteristics and influencing factors in the bifurcation area of coal seam. It can explore the initiation, expansion and evolution mechanism of cracks inside coal or rock from a micro-scopic perspective, making the instability process more transparent. Moreover, ultrahigh repeatability makes it convenient to study the precursory characteristics and influencing factors of rockburst with the control variable method.

Based on this, with the help of the UDEC, this paper constructs a numerical model of the "coal-rock parting-coal" combined structure (CRCS). This paper studies the failure and instability characteristics of the CRCS from a micro-scopic perspective and explores the factors influencing the failure and instability of the CRCS. Based on the secondary development of the "FISH" program, a crack tracking and identification method was developed, and the crack expansion law and energy evolution characteristics of each unit were studied. At the same time, the main factors affecting and precursor characteristics are identified. This study is helpful for enriching the theory of dynamic instability of coal and rock and has theoretical guiding significance for preventing rockburst accidents caused by structural failure and instability in the bifurcation area of coal seam.

## 2 Model parameters

### 2.1 UDEC calculation principle

**Fig 1:**
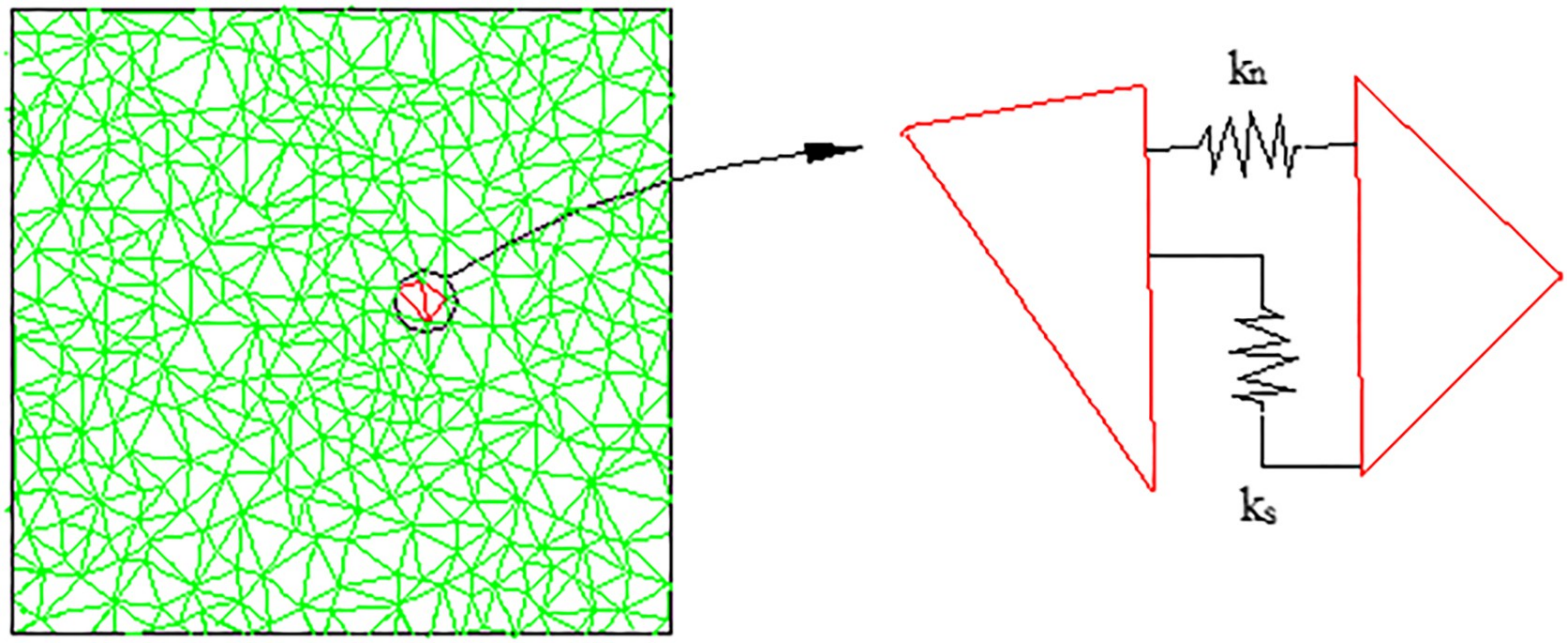
UDEC block contact model.

Rock mass is a combination of a series of blocks and joints in the UDEC [28]. Under the action of dynamic and static load, the block can undergo elastoplastic deformation, the joint will experience shear and tensile failure, and the block and joint follow their own constitutive criteria. The UDEC block contact model is shown in Fig 1.

The deformation characteristics of the block are characterized by the bulk modulus (K) and shear modulus (G), which are calculated by Formulas (1) and (2) [28].

$$K=\frac{E}{3(1-2\nu )}$$ (1)

$$G=\frac{E}{2(1+\nu )}$$ (2)

where *E* and *ν* are the Young’s modulus and Poisson’s ratio, respectively.

The normal stiffness and tangential stiffness of the joint are calculated by Formula (3) and formula (4) [28].

$$k_{n}=n[\frac{K+(4/3)G}{\Delta Z_{\min}}]\;1\leq n\leq 10$$ (3)

$$k_{s}=0.4k_{n}$$ (4)

where *k*_*n*_ is the normal stiffness of the joint, *k*_*s*_ is the tangential stiffness of the joint, *K* and *G* are the bulk modulu and shear modulu of the block, respectively, and ΔZ_min_ is the minimum side length of the contact normal element.

The strength of the joint surface is defined by the frictional angle *φ*, cohesion *c* and tensile strength *σ*_*t*_ [28].

Normal direction:

$$\Delta \sigma_{n}=-k_{n}\Delta u_{n}$$ (5)

where Δ*σ*_*n*_ is the normal effective stress increment and Δ*u*_*n*_ is the normal displacement increment. When the tensile stress of the joint surface exceeds *σ*_*t*_, that is, Δ*σ*_*n*_ = 0, tensile failure occur in the normal direction of the joint surface.

In the shear direction [28]:

$$|\tau_{s}|\leq c+\sigma_{n}\tan\phi =\tau_{\max}$$ (6)

Then,

$$\Delta \tau_{s}=-k_{s}\Delta u_{s}^{e}$$ (7)

Otherwise,

$$|\tau_{s}|\geq \tau_{\max}$$ (8)

Then,

$$\tau_{s}=sign(\Delta u_{s}^{e})\tau_{\max}$$ (9)

where $\Delta \mu_{s}^{e}$ is the elastic component of the incremental shear displacement, *u*_*s*_ is the tangential displacement, and *τ*_max_ is the maximum shear strength of the joint surface. If |*τ*_*s*_|≥*τ*_max_, the joint surface experience shear failure in the tangential direction.

### 2.2 Parameter calibration

The mechanical parameters required by the UDEC numerical model are two kinds of meso-scopic parameters, namely, the block and the interface, which cannot be obtained directly through laboratory mechanical test. The mechanical parameters obtained by numerical simulation should be compared with the macro-scopic mechanical parameters obtained by the experiment, and the meso-parameters should be modified according to the comparison results until the values are in accordance with the laboratory results. Therefore, based on laboratory uniaxial compression experiment and Brazilian splitting experiment, the meso-parameters of coal and rock parting are calibrated. The selected coal and rock parting were sampled from the same borehole in the 1st mining area of the Zhaolou Coal Mine. The calibration results are shown in Tables 1 and 2, respectively.

**Table 1:** Meso-parameters of the block after calibration.

Material	Density/(kg/m^3^)	K/GPa	G/GPa
Coal	1400	2.87	1.48
Rock parting	2430	5.47	2.82

**Table 2:** Meso-parameters of interface after calibration.

Material	*k*_*n*_/(GPa/m)	*k*_*s*_/(GPa/m)	Coh./MPa	Fri./°	Ten. strength/MPa
Coal	5966	2386.4	1.43	24	0.96
Rock parting	12673	5069.2	4.02	30	1.83

### 2.3 Numerical model

**Fig 2:**
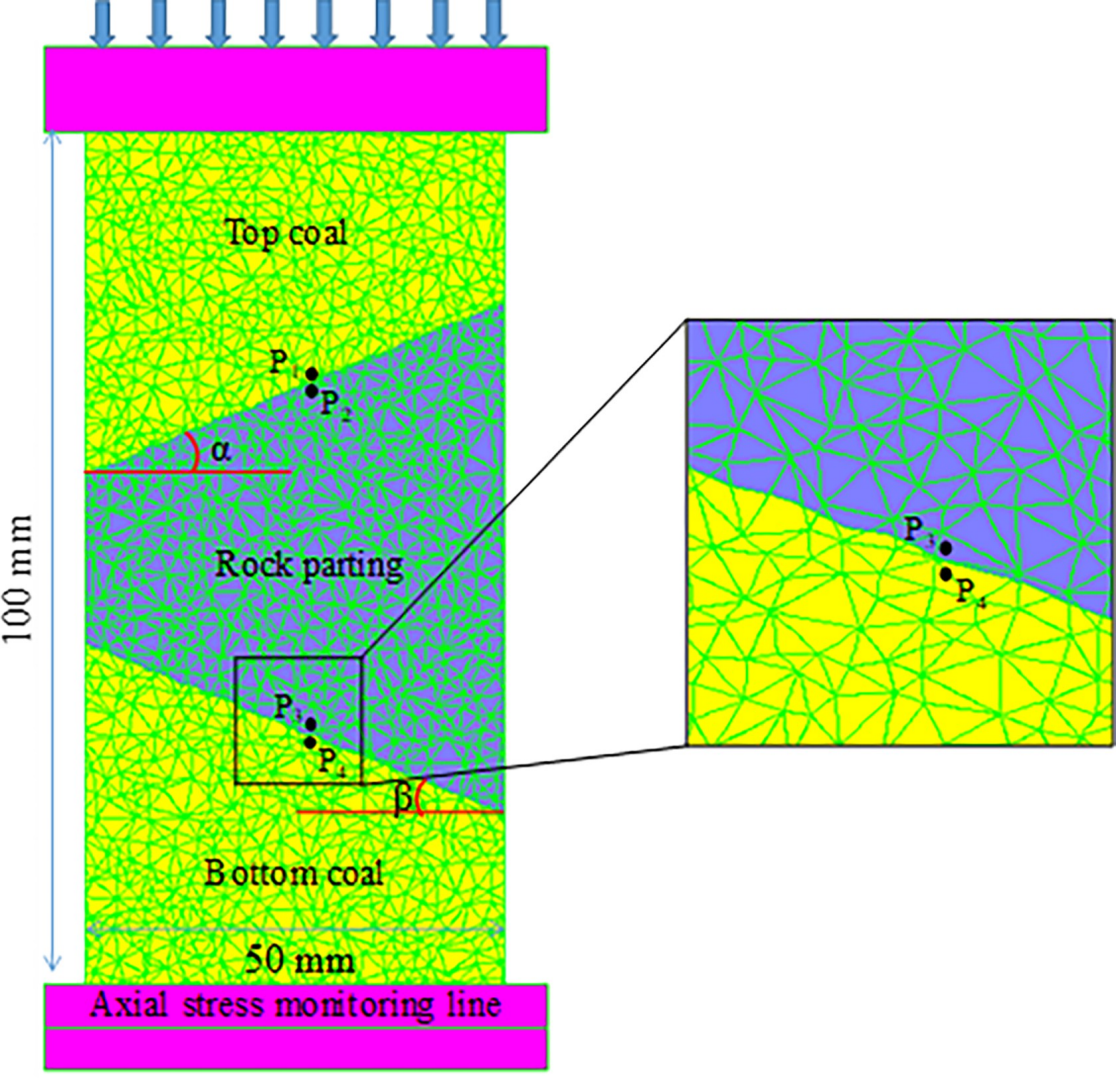
Numerical model of the CRCS.

At 2:49 on July 29, 2015, a rockburst accident occurred at the 1305 working face of the Zhaolou Coal Mine of Yanmei Heze Chemical Co., Ltd. The energy monitored by the Seismological Observation System (SOS) monitoring system was only 2.5×10^6^ J, and no warning information was detected before the rockburst occurred. However, rockburst can be very destructive, resulting in 3 people being injured (1 seriously injured, 2 lightly injured) and a direct economic loss of 938,700 yuan. An accident was identified as slip and instability of rock parting under the action of high static stress on the working face of an island [29]. Therefore, this paper uses the micro-scopic parameters in Table 1 to establish the CRCS ternary series combination structure model, and the model is shown in Fig 2. The diameter and height of the model are 50 mm and 100 mm, respectively, and the left and right widths of the central rock parting are 20 mm and 60 mm, respectively. The wave shapes of the upper and bottom discontinuities are set at the same angle, and the joint roughness coefficient (JRC) is 5.34 and 5.16, respectively. Monitoring points P_1_ and P_2_ are distributed in the coal and rock parting along the upper contact surface. Points P_3_ and P_4_ are located in the rock parting and coal of the bottom discontinuities, respectively. During loading, the stress, strain and slip of the model are recorded by the “Hist” function. The “Fish” program is used to record the shear and tensile cracks inside coal and rock parting, and the energy accumulation and release of the model are monitored by the “Config” function.

## 3 Failure and instability characteristics of CRCS

### 3.1 Evolution characteristics of crack development

**Fig 3:**
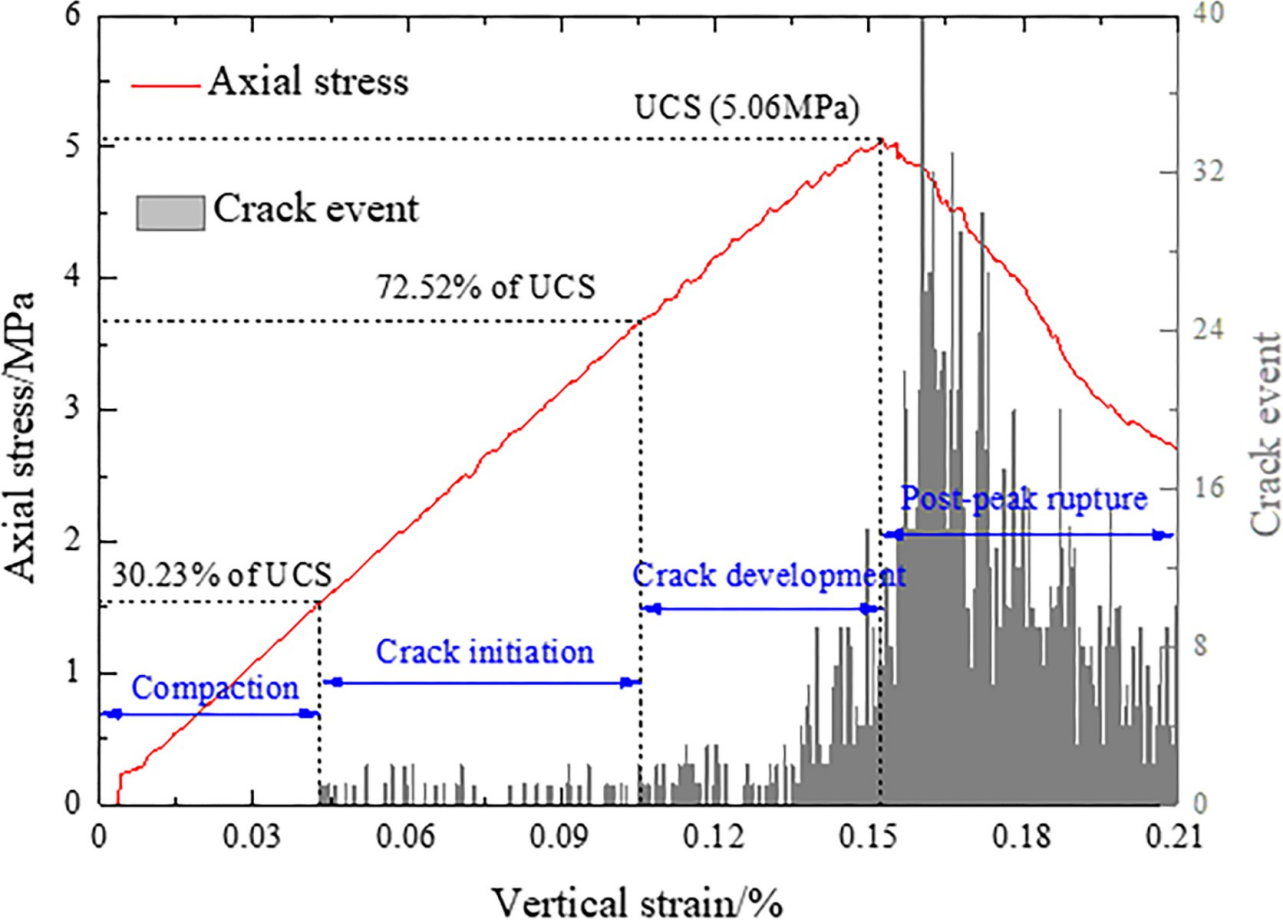
Axial stress and crack events versus axial strain per 100 steps.

Fig 3 shows the evolution of crack development during fracture, slip and instability of the model. According to the relationship between stress and crack evolution, the crack development process can be divided into four stages: (1) Compaction stage: no cracks are formed, the stress linearly increases, and the elastic energy accumulates gradually; (2) Crack initiation stage: a small number of cracks are generated, and the stress fluctuates slightly with cracks. The initiation process includes the cycle of “stable—fracture—stable”. (3) Crack developmental stage: the stress fluctuates obviously, the number of cracks increases rapidly, the stability time of the cracks decreases gradually, and the development time of the cracks increases gradually. When the stress reaches the peak, the stability time of the cracks decreases to 0. (4) Post-peak rupture stage: the number of cracks reaches peak and begins to connect and form macro-cracks. After that, the stress gradually decreases, and the crack development gradually weakens. The failure process of the combined samples also includes the initiation, expansion and penetration of cracks.

**Fig 4:**
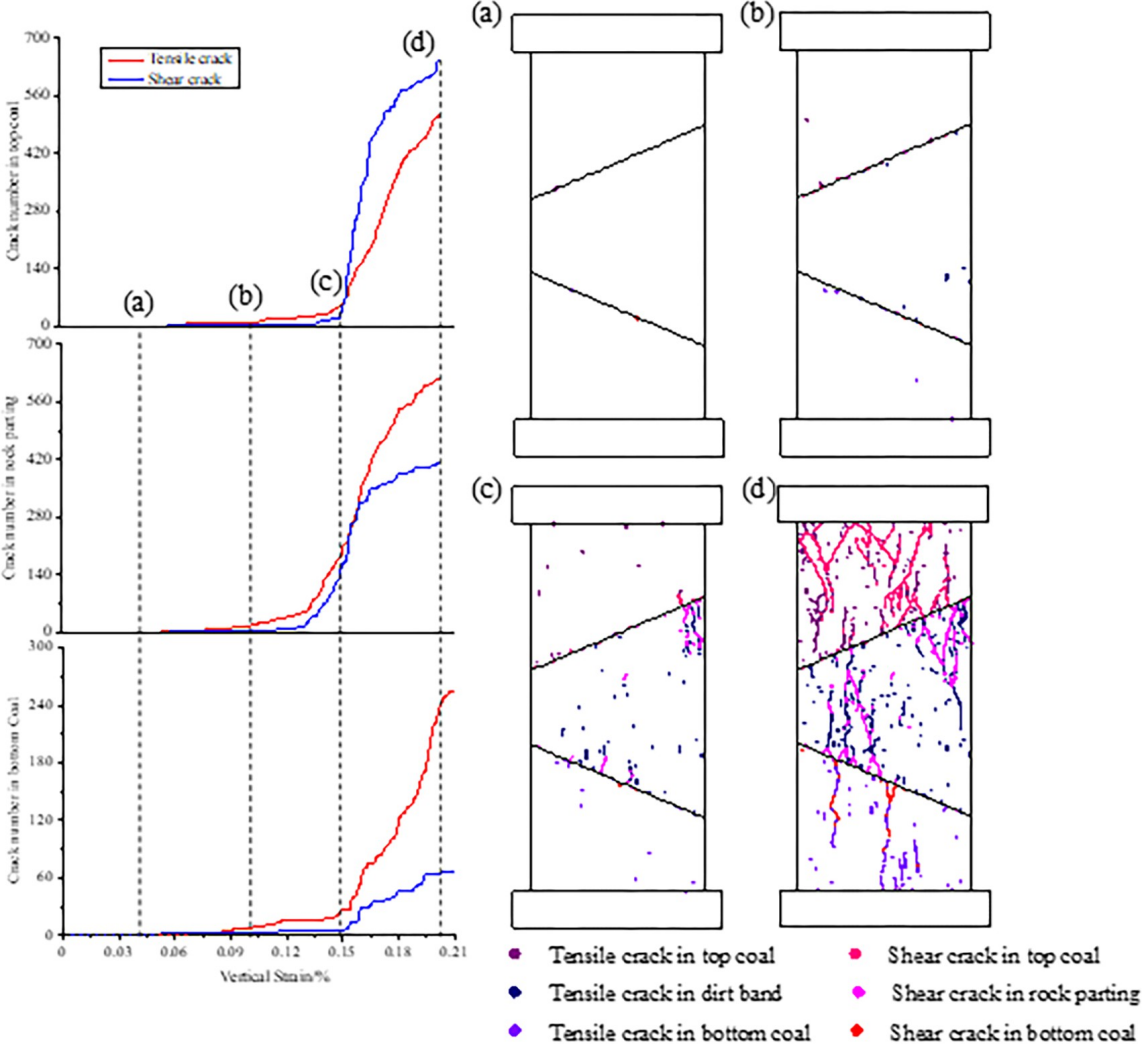
Type, number and location of cracks inside the coal and rock parting.

Fig 4 shows the type, number and location of cracks inside the coal and rock parting during fracture, slip and instability. Points (a), (b), (c) and (d) are the boundary points of the four stages shown in Fig 4. The crack initiation threshold is the first stress point of joint failure, and the crack damage threshold is the stress point of rapid growth of joint failure in rock and coal mass [30]. The crack initiation threshold is approximately 30% of UCS, as shown at point (a). The initial crack is tensile and is first generated along the discontinuities. This is because the strength of the contact surface is weaker than that of the coal or rock parting. The crack damage threshold is approximately 72% of UCS, as shown at point (b). The cracks develop rapidly, tensile cracks gradually form inside the coal and rock parting, and there is aggregation of shear cracks on the discontinuities. The crack initiation threshold and crack damage threshold are basically consistent with the results of Hoek and Martin’s study [30]. This shows that the initial failure process of the combined structure is basically the same as that of single coal or rock mass. Point (c) corresponds to the peak stress. During this stage, the stress increases rapidly, and the number of cracks increases significantly. The UCS of rock parting is much larger than that of coal, but rapid crack growth first occurs in the rock parting. It can be seen from the crack locations in subgraph (c) that many cracks are formed perpendicular to the discontinuities and further extend into the coal and rock. This fully indicates that stress concentration easily occurs along the discontinuities, thus triggering fracture and slip. This is significantly different from the whole penetration fracture comparison of single coal or rock-coal combined mass [31]. This shows that the slipping action of the contact surface can change the fracture evolution characteristics of the combined structure and subsequently affect the fracture effect of the combined structure. After point (c), the cracks coalesce and thus form macro-crack, and the number of new cracks gradually decreases from top to bottom. In addition, the top coal is mainly characterized by shear failure, and the failure of the rock parting and bottom coal is mainly caused by tensile cracks.

### 3.2 Slip characteristics of the discontinuities

**Fig 5:**
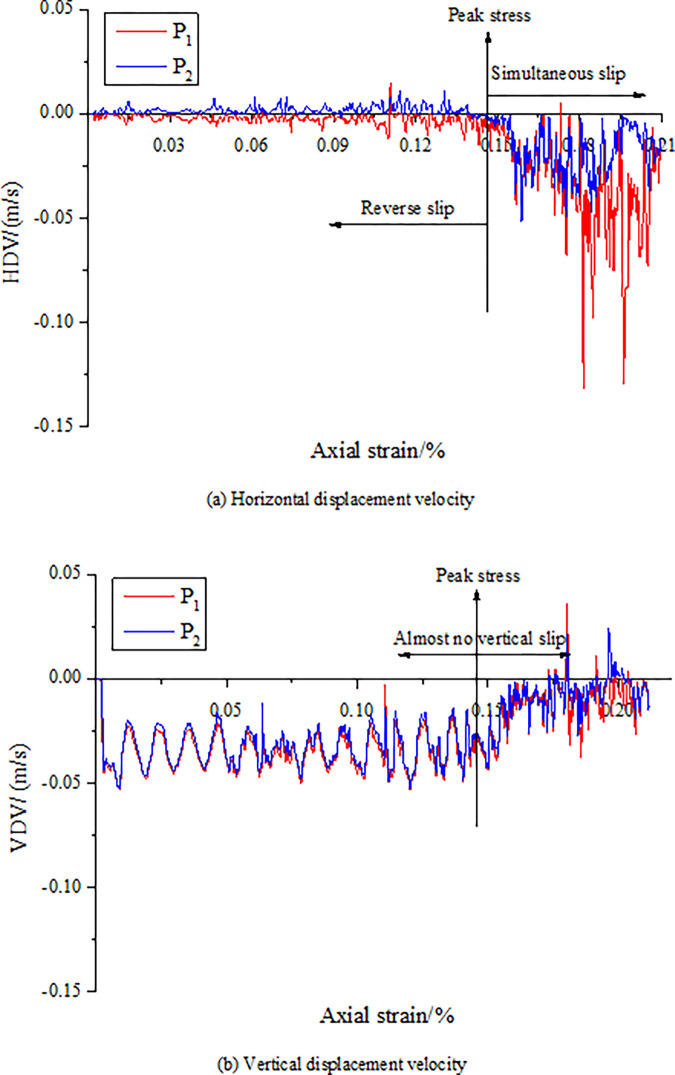
Horizontal and vertical displacement velocities of the points P_1_ and P_2_. (a) Horizontal displacement velocity. (b) Vertical displacement velocity.

**Fig 6:**
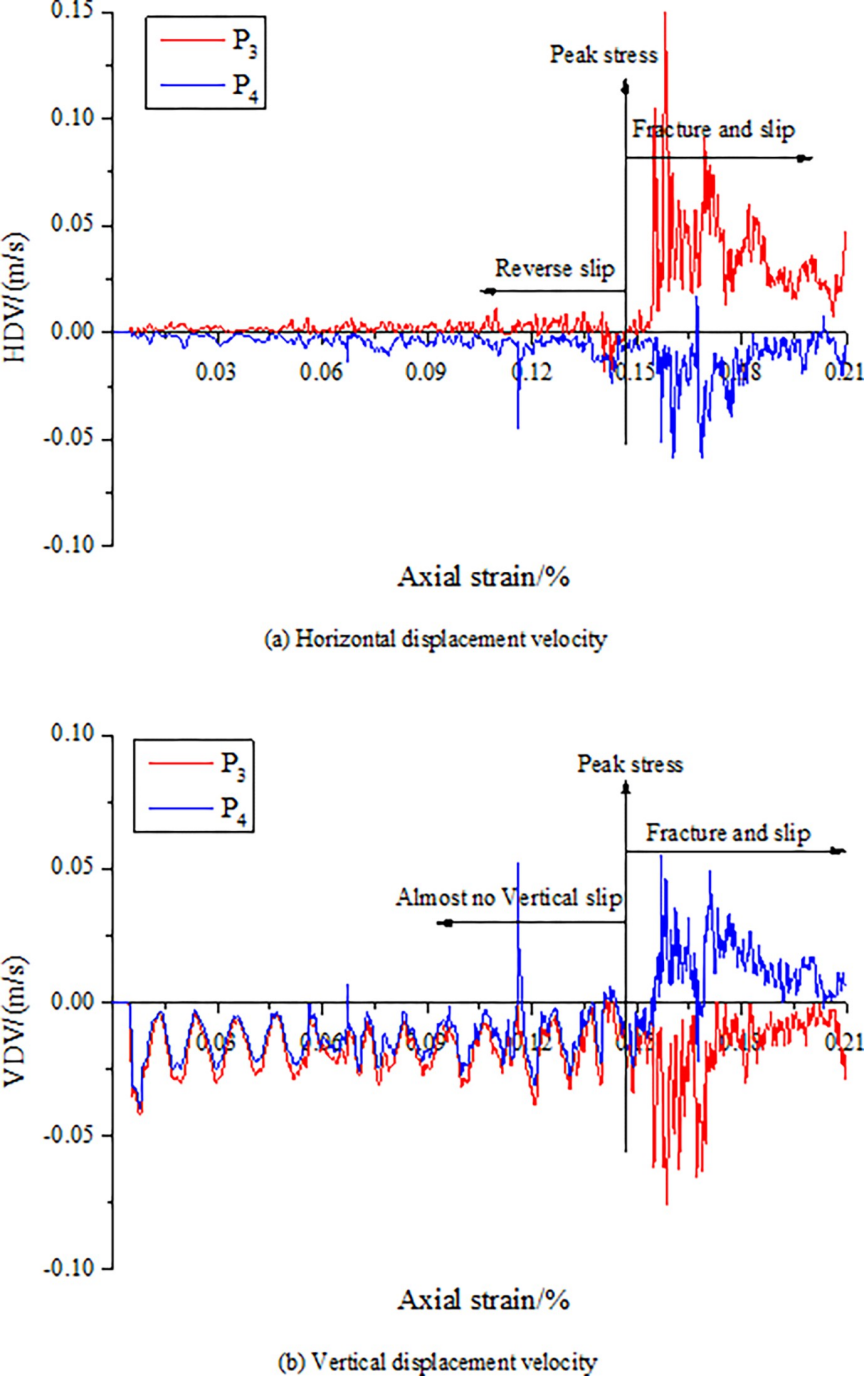
Horizontal and vertical displacement velocities of the points P_3_ and P_4_. (a) Horizontal displacement velocity. (b) Vertical displacement velocity.

Under the action of stress, cracks occur along the discontinuities of CRCS, accompanied by slip. To further verify the slip characteristics of the discontinuities, the velocities of the horizontal and vertical displacements are recorded at four monitoring points arranged in the coal and rock parting, as shown in Figs 5 and 6.

Fig 5 shows that the vertical displacement velocities (VDV) of points P_1_ and P_2_ are basically the same, but there is a significant difference between them in term of the horizontal displacement velocity (HDV). Before reaching the peak stress, the HDV direction of the rock parting (positive direction of the X-axis) is opposite to that of the coal (negative direction of the X-axis). After that, the displacement direction of the rock parting reverses, and the velocity is obviously smaller than that of the coal. This shows that before and after the peak, there are two different slip phenomena on the top coal and rock contact surface. Before the peak, under the action of axial stress, the coal and rock parting slips and misplaces along the two contact surfaces in opposite direction. After the peak, the rock parting slip and dislocate in the same direction under the action of the friction force of the coal slip.

From Fig 6, the HDV of points P_3_ and P_4_ are always reversed, and the velocity of the rock parting is greater than that of the coal. The VDV significantly changes after reaching the peak stress, and the direction of the rock parting (negative direction of the Y-axis) is opposite to that of the coal (positive direction of the Y-axis). In addition, the VDV of points P_1_ and P_2_ are also along the positive direction of the Y-axis, which may be due to elastic deformation restoration caused by the release of elastic energy in the coal after the coal and rock parting becomes unstable. Therefore, abnormal changes in the HDV and VDV can be regarded as precursor characteristics.

### 3.3 Energy evolution characteristics

**Fig 7:**
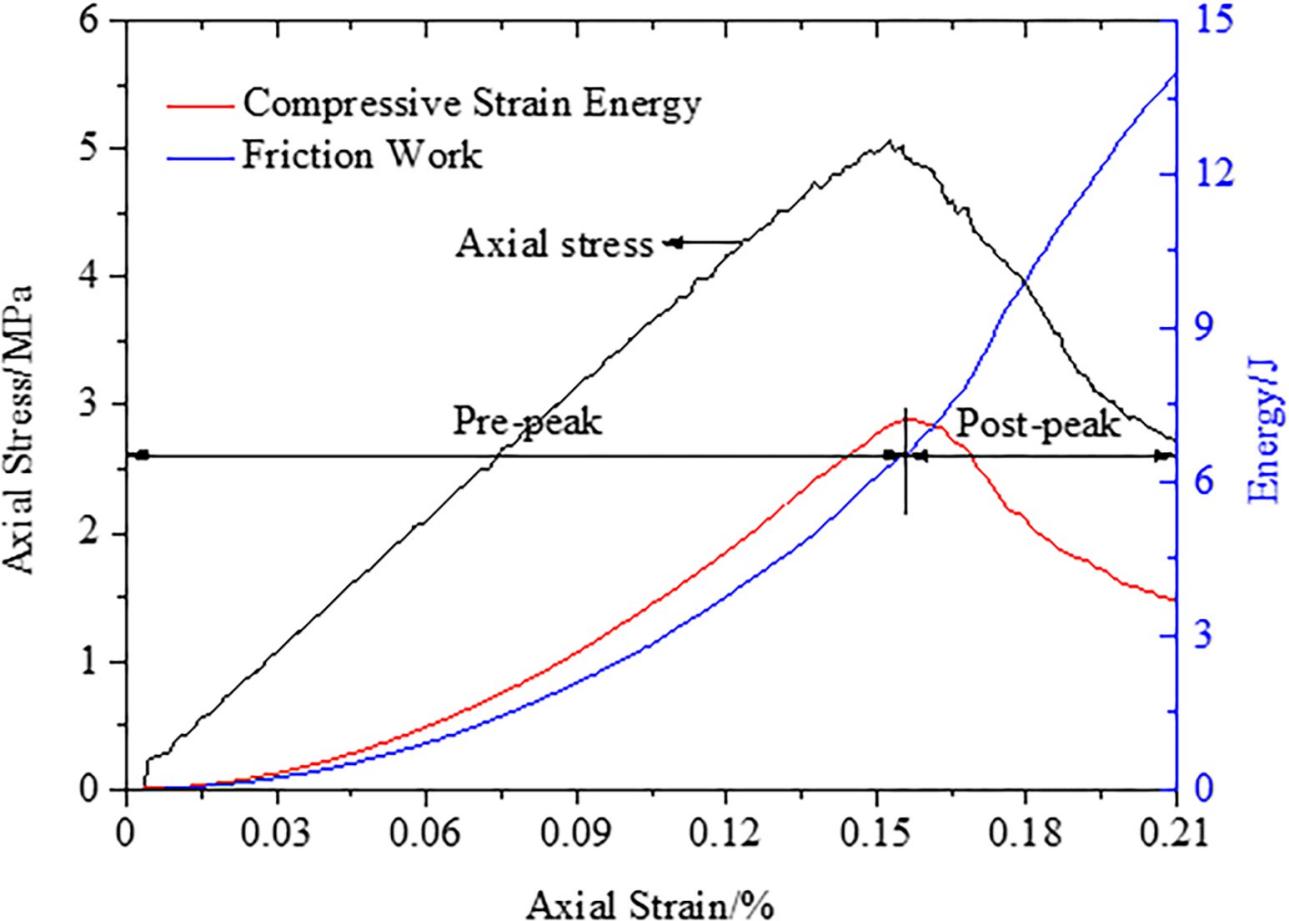
Energy accumulation and dissipation of the CRCS during loading until final instability.

Fig 7 shows the energy accumulation and dissipation of the CRCS during the whole process of deformation, fracture and slip until final instability. The compressive strain energy increases gradually before reaching the peak stress and then decreases rapidly. The energy accumulation and release process is consistent with the change in stress. Before reaching the peak stress, the frictional work basically corresponds to the strain energy accumulation. With the accumulation of energy, shear and tensile cracks gradually initiate. Simultaneously, the cracks increase the slip surface between the coal and rock parting, and thus, the friction gradually increases. In the post-peak rupture stage, the friction further increases due to the rapid development of cracks, and the development reaches peak. Therefore, the frictional slip and fracture of the discontinuities promote each other. Friction accelerates the formation of cracks, and conversely, the newly formed cracks increase the frictional slip surface and accelerate the failure of CRCS.

**Fig 8:**
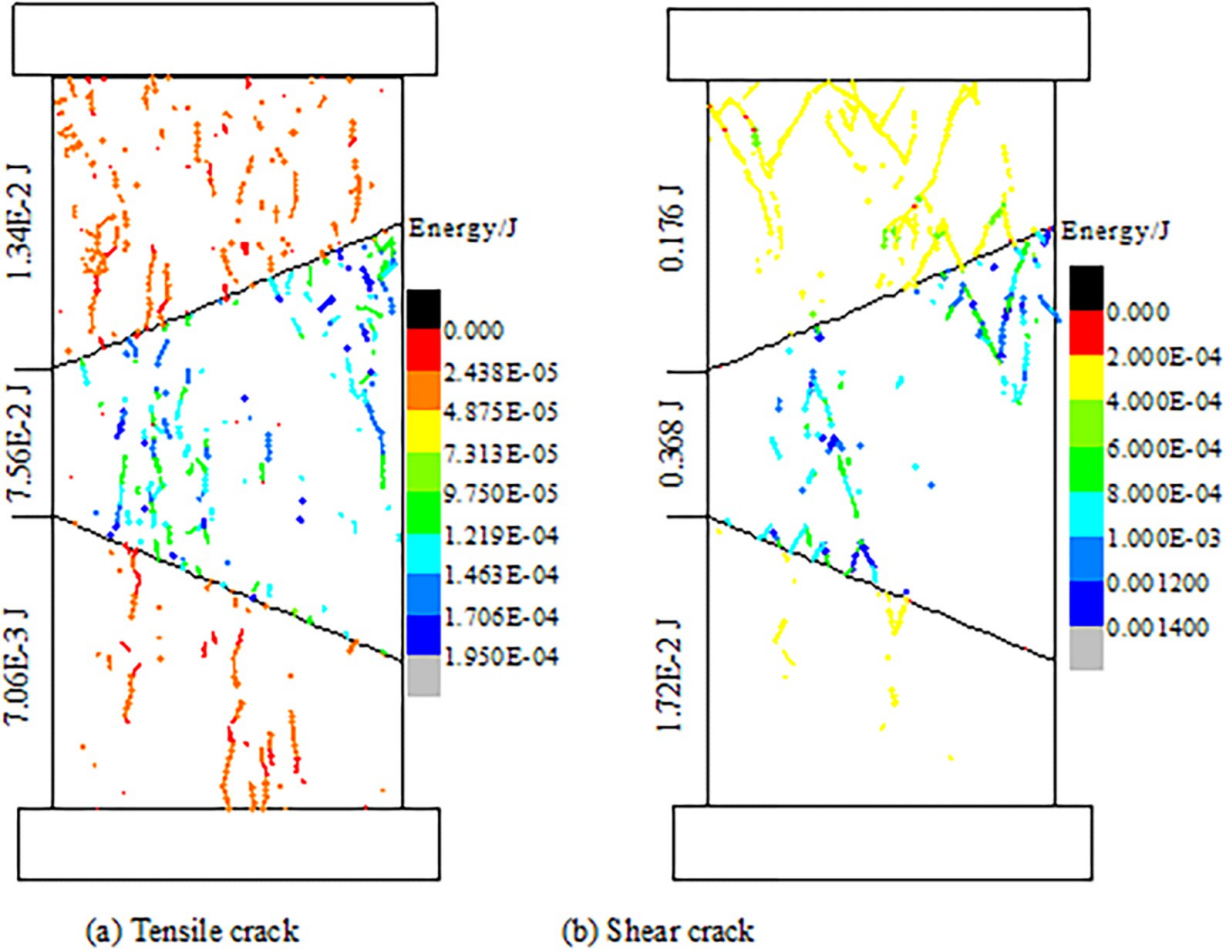
Fracture dissipation energy of the coal and rock parting of CRCS. (a) Tensile crack. (b) Shear crack.

Fig 8 shows the fracture dissipation energy of the coal and rock parting of CRCS. The energy dissipation of rock fracture and single joint fracture is significantly greater than that of coal fracture, and cracks in rock parting develop faster. These phenomena fully indicate that the energy accumulated in the rock parting is greater than that in the coal. Moreover, the total energy consumed by shear cracks and by single joint shear cracks is obviously greater than that consumed by tensile cracks. This shows that the failure modes of the CRCS are first tensile cracking and second shear cracking.

In summary, once the discontinuities generate unstable slip, a large amount of strain energy will be released. The strain energy can easily induce dynamic disasters in the vicinity of high stress concentration in coal and rock masses, such as rockburst and tremors. There are obvious precursors prior to failure and instability of CRCS. For example, 1) the cycle time of “stable—fracture—stable” fracture development decreases, and cracks develop rapidly; 2) there are abnormal changes in the HDV and VDV; and 3) obvious slip of discontinuities and rapid energy accumulation in rock parting occur. These precursors can provide early warning for geological dynamic disasters induced by slip and instability of CRCS.

## 4 Influence of coal and rock parting parameters

### 4.1 Inclination angle effect

**Fig 9:**
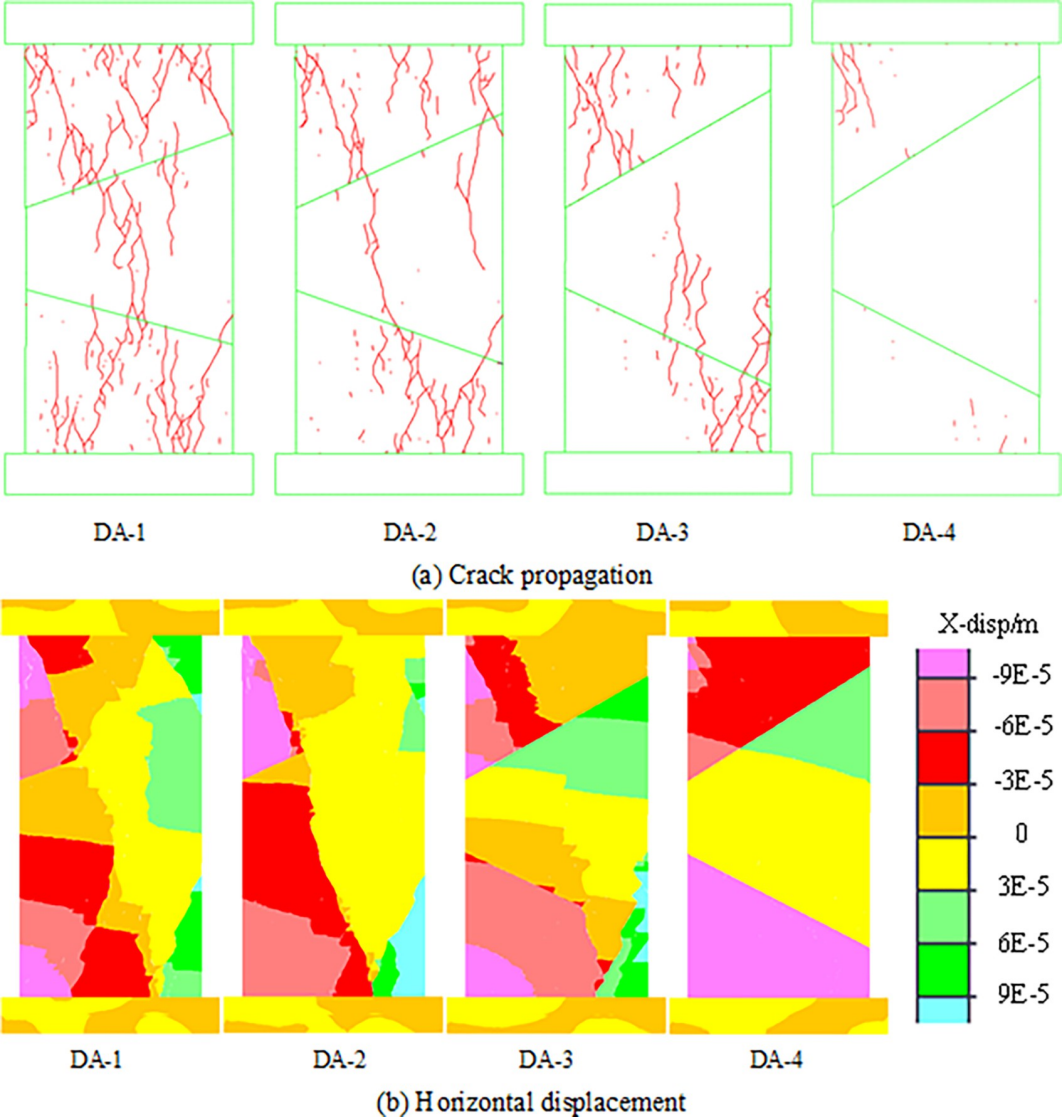
Failure and instability forms of models with different inclination angles. (a) Crack propagation. (b) Horizontal displacement.

Due to the change in geological conditions, the inclination angle between the rock parting and coal seam will change irregularly, and the change in inclination angle has an important effect on the instability characteristics of the combined models [32, 33]. By constantly changing the inclination angle of contact surface, the effect of inclination angle on the overall instability characteristics of combined models is studied. The combined models with different inclination angles are shown in Table 3. The crack propagation and horizontal displacement of the combined models with different inclination angles are shown in Fig 9.

**Table 3:** Combined models with different inclination angles.

Model	α/°	β/°
DA-1	20	15
DA-2	25	20
DA-3	30	25
DA-4	32.5	27.5

From Fig 9, neither the combined model DA-1 nor DA-2 produces an obvious slip phenomenon, and the fractures are mainly vertically penetrating fractures. This indicates that the instability of the combined model is characterized by fracture instability when the inclination angle of the contact surface is small. With increasing inclination angle of the contact surface, slip instability occurs along the contact surface of the coal and rock. The fracture development of the combined model changes from vertical through fracture to shear slip fracture perpendicular to the contact surface, and the model instability also changes from fracture instability to slip instability. Different from the slip instability of single contact surface (model DA-3), the slip phenomenon occurs on both the upper and lower contact surface during the instability of model DA-4. This indicates that with increasing inclination angle of the contact surface, the slip instability of the coal and rock parting combined structure changes from single contact surface to double contact surface.

**Fig 10:**
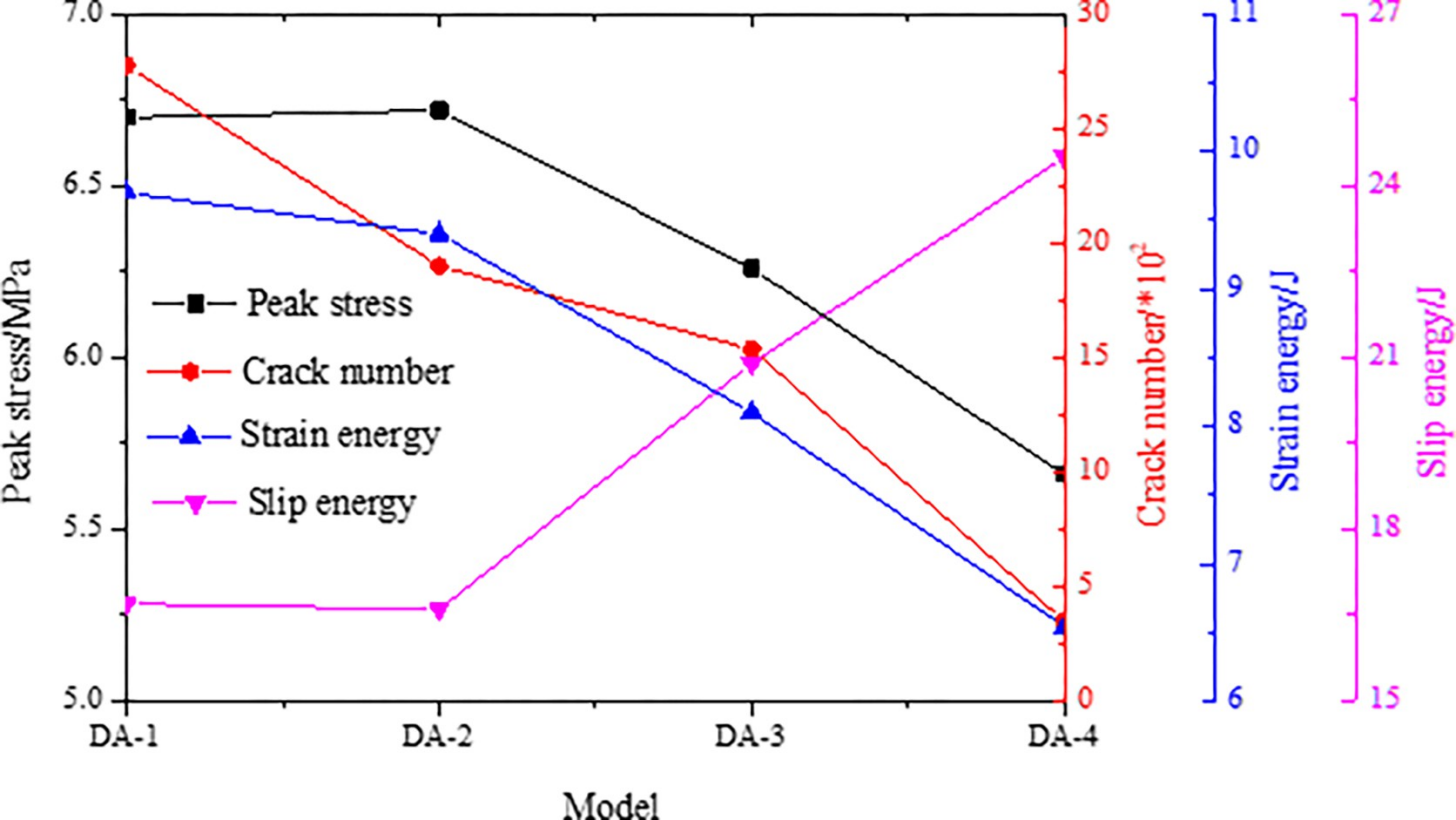
Parameter evolution characteristics of models with different inclination angles.

To explore the failure and instability characteristics of the combined model with different inclination angles, the peak stress, number of cracks, strain energy and slip dispersion during the instability process are monitored, and the results are shown in Fig 10.

When the inclination angle of the contact surface is small, the monitoring data of each parameter of the combined models DA-1 and DA-2 are basically consistent, which indicates that the change in the inclination angle of the contact surface has basically no effect on the instability of the combined models. However, when the inclination angle of the contact surface exceeds the friction coefficient, the peak strength, number of cracks and strain energy of the combined model decrease rapidly, and the combined model changes from fracture instability to slip instability. These changes reduce the model’s bearing capacity and stability and make the model more prone to overall structural instability. The slip energy increases rapidly after model instability, which indicates that the accumulated energy in the model is released rapidly through the slip form, and the instability of the model is characterized by low intensity and high energy release. Therefore, rockburst of structural instability is more difficult to monitor in the bifurcation area, and rockburst disaster caused by such instabilitie is stronger than those caused by whole fracture and instability. This is basically consistent with the scene of the "7.29" rockburst accident in the Zhaolou Coal Mine.

### 4.2 Strength effect of rock parting

**Fig 11:**
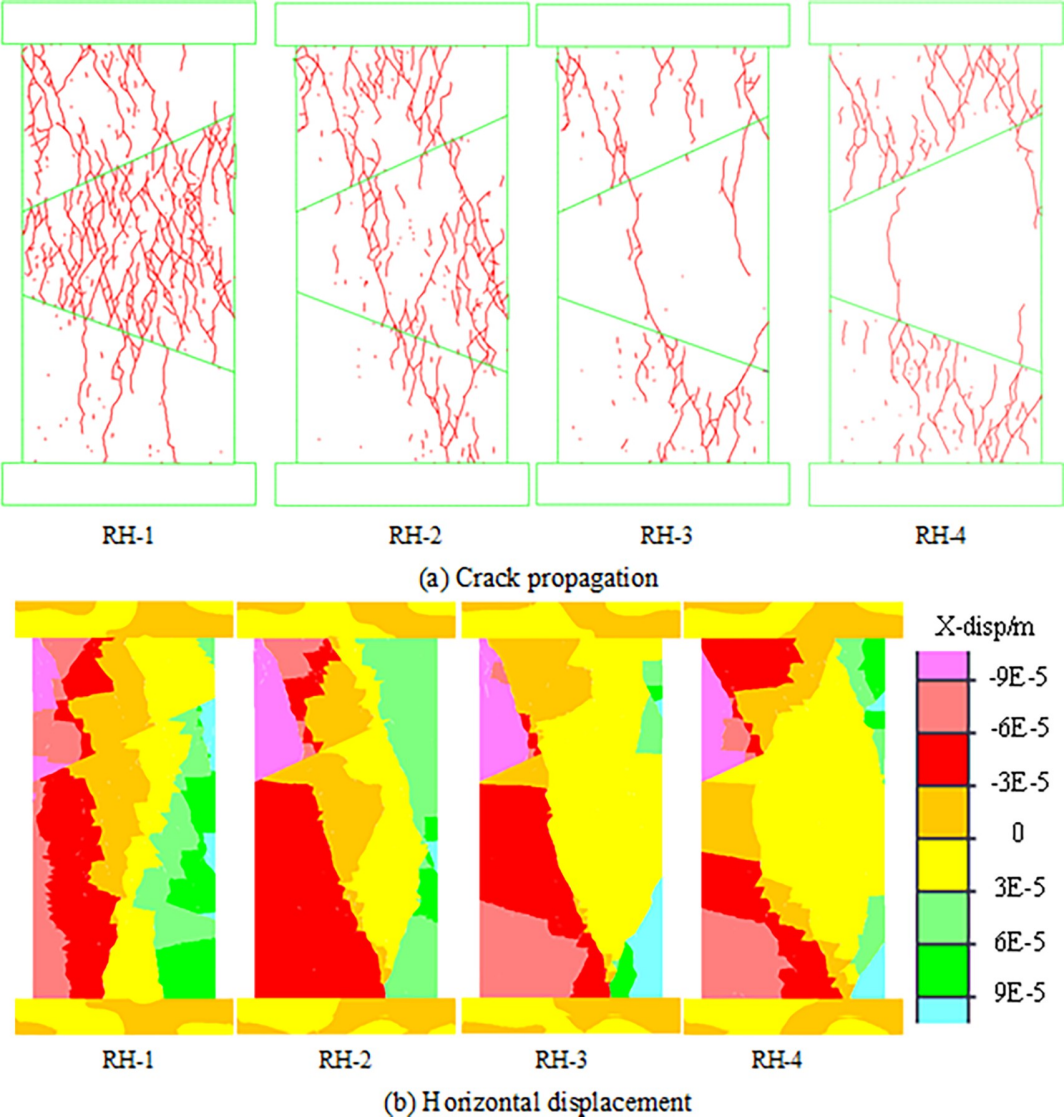
Failure and instability forms of models with different strength of rock parting. (a) Crack propagation. (b) Horizontal displacement.

The rockburst tendency of coal and rock mass is controlled by the strength of the material, and hard coal and rock mass are more likely to accumulate elastic energy, after which sudden brittle failure occurs, resulting in rockburst accidents [34]. Therefore, the change in the rock parting strength also has a very important effect on the rockburst characteristics of the combined model. To explore the influence of the strength of the rock parting on the failure and instability characteristics of the combined structure, model DA-2 is selected as the research model to study the effect of the strength of the rock parting by constantly changing the parameters of the rock parting. Table 4 shows the combination models for different strengths of rock parting. The strength of the rock parting in models RH-1 and RH-2 is lower than that of the coal, and the strength of the rock parting in the simulated project site is soft, while the strength of the rock parting in models RH-3 and RH-4 is greater than that of the coal, and the strength of the rock parting in the simulated project site is hard. The crack propagation and horizontal displacement of the combined models with different rock parting strength are shown in Fig 11.

**Table 4:** Combination models for different strength of rock parting.

Model	Coh./MPa	Ten. strength/MPa
RH-1	1.56	0.68
RH-2	2.11	0.85
RH-3	3.42	2.21
RH-4	5.44	3.67

According to the research in Section 3.1, the instability of model DA-2 is manifested as fracture instability. However, when the strength of the rock parting is less than that of the coal, the coal and rock parting of model RH-1 has an obvious slip phenomenon, and the form of instability is single contact slip instability. With increasing strength of the rock parting, the slip phenomenon of the upper contact surface gradually becomes less obvious, and the model returns to the fracture instability state. This shows that the change in coal and rock parting strength directly affects the fracture instability characteristics of the combined model and indirectly affects the slip instability characteristics of the combined model. When the strength of the rock parting is less than the strength of the coal, the fracture development is mainly concentrated in the rock parting. When the strength of the rock parting exceeds the strength of the coal, the fracture development is mainly concentrated in the coal, and the change in the fracture instability caused by the difference in the coal and rock parting strength is controlled by the material properties with low strength. When the strength of the rock parting is small, the rock parting will be seriously broken. The broken rock parting will cause the friction performance of the contact surface to decrease, which causes the combined model to change from fracture instability to slip instability.

**Fig 12:**
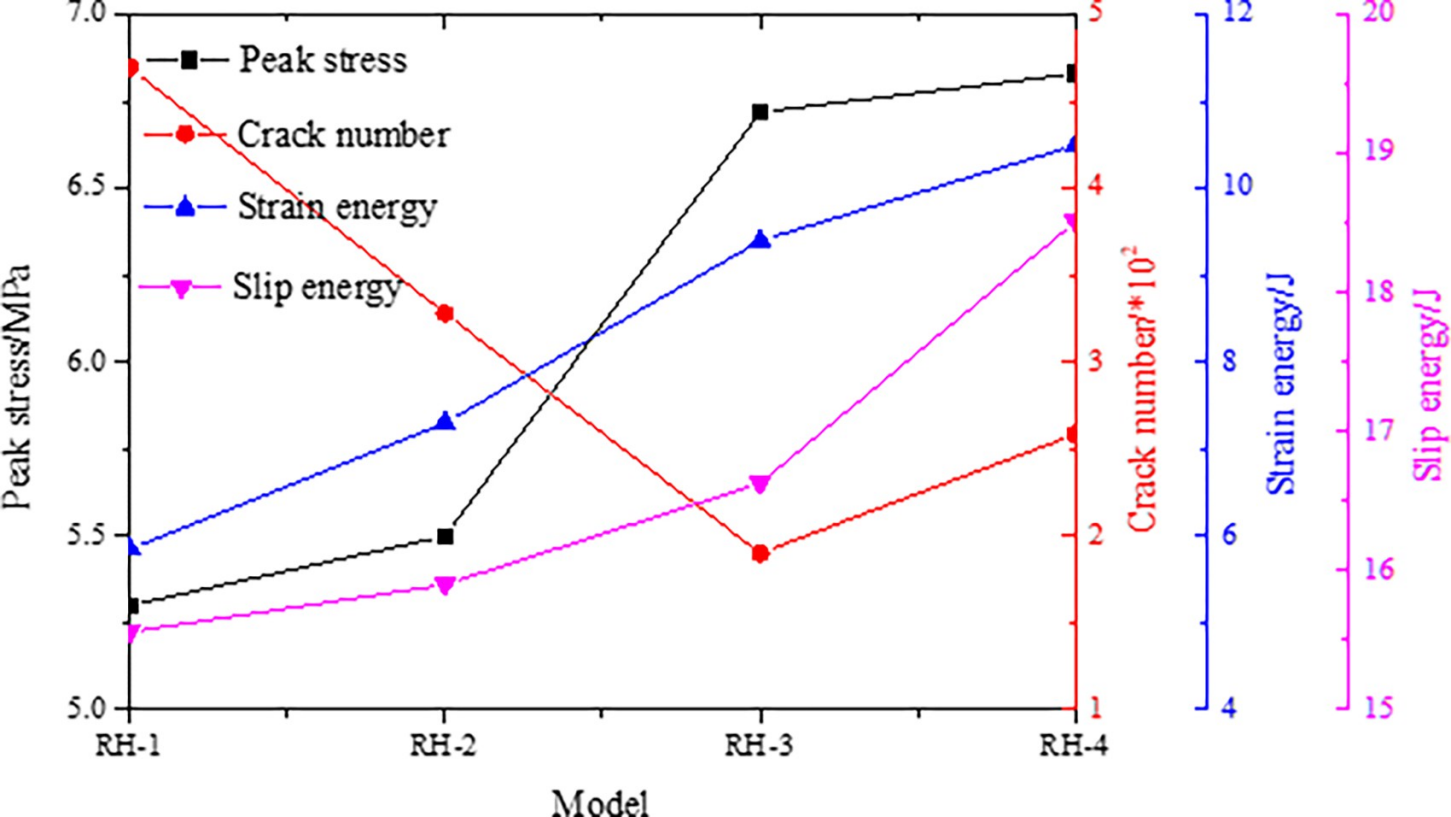
Parameter evolution characteristics of models with different strength of rock parting.

**Fig 13:**
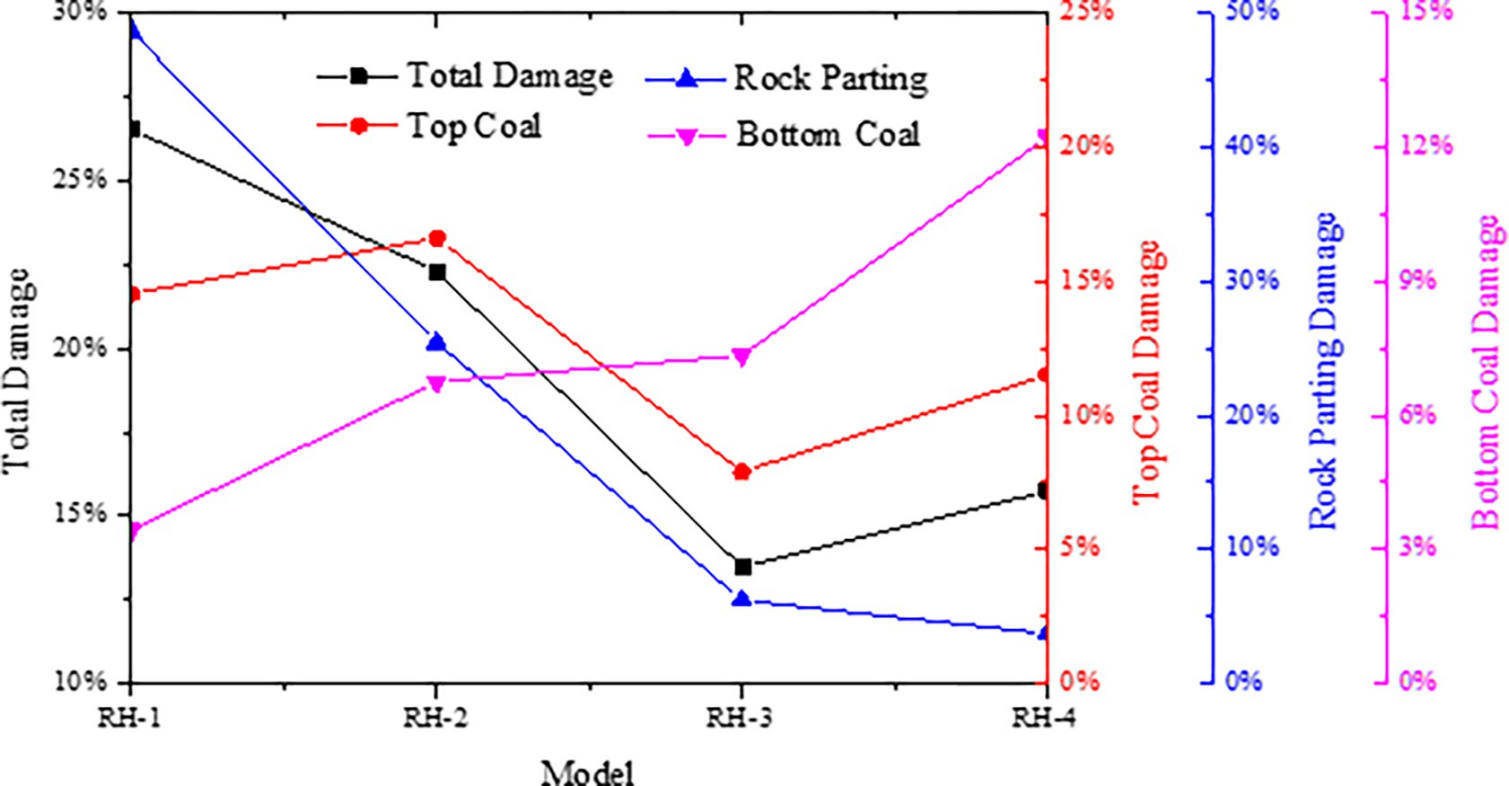
Damage degree of each unit in the models with different strength of rock parting.

The peak stress, number of cracks, strain energy and slip dispersion during the failure process of the combined models with different strength of rock parting are monitored. The monitoring results are shown in Fig 12, and the damage degree of each unit of the models is shown in Fig 13.

The peak instability strength of the combined model increases with increasing intensity of the rock parting. The number of cracks gradually decreases with increasing strength of the rock parting when the strength of the rock parting is less than that of the coal. When the strength of the rock parting is greater than the strength of the coal, the number of cracks increases gradually with increasing strength. The greater the strength of the rock parting is, the greater the strain energy and slip energy stored in the combined model, and the greater the rockburst risk. When the strength of the rock parting is small, the instability of the combined model shows that the rock parting breaks first and the coal slips later. When the strength of the rock parting is large, the instability of the combined model shows that the coal breaks first and the rock parting slips later. Therefore, according to the strength difference between coal and rock parting, the fracture and slip instability of the coal and rock parting can be prevented in advance at the project site.

### 4.3 Thickness effect of rock parting

**Fig 14:**
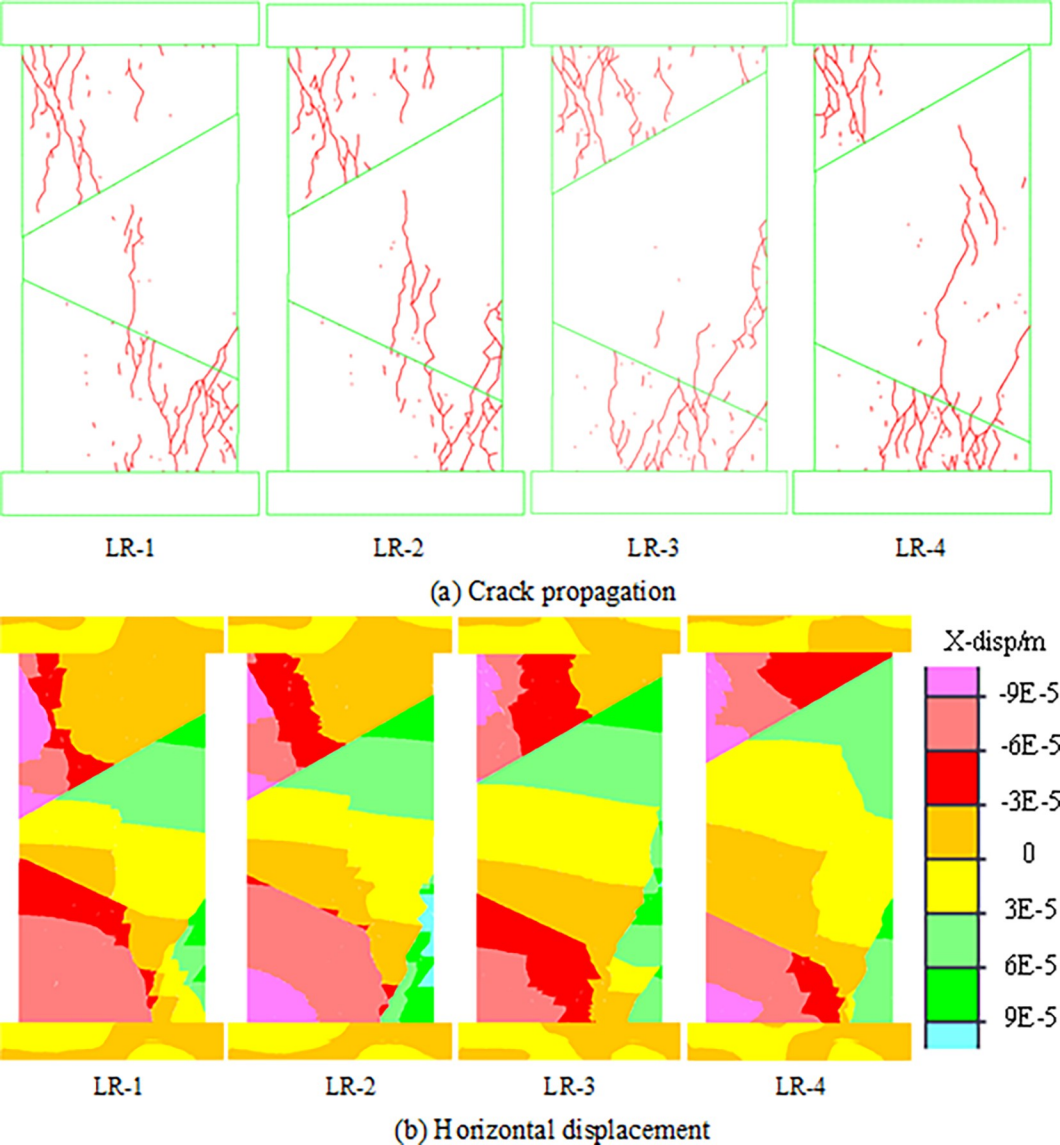
Failure and instability forms of models with different thicknesses of rock parting. (a) Crack propagation. (b) Horizontal displacement.

The change in coal thickness is also an important index for evaluating rockburst risk. Generally, the greater the thickness of coal is, the greater the rockburst risk [35]. A change in the rock parting thickness can cause a change in the seam thickness and subsequently affect the rockburst risk in the bifurcation area of coal seam. Therefore, model RH-3 is selected as the research model to explore the effect of the change in thickness of the rock parting on the failure and instability characteristics of the combined structure. By keeping the inclination angles of the upper and lower contact surface unchanged and constantly changing the thickness of the rock parting, the influence on the mechanical properties of the combined models can be studied. The combined models with different rock parting thickness are shown in Table 5. The crack propagation and horizontal displacement of the combined models with different rock parting thickness are shown in Fig 14.

**Table 5:** Combination models of different thickness of rock parting.

Model	Left/mm	Right/mm
LR-1	10	62.2
LR-2	20	72.2
LR-3	30	82.2
LR-4	40	92.2

Cracks gradually evolve from the middle of the model to the edge with increasing thickness of the rock parting. An increase in the thickness of the rock parting causes a concentration of stress at the edge of the coal and rock parting, which causes cracks to expand to the edge of the combined model. Therefore, change in the thickness of the rock parting can cause change in the fracture instability characteristics of the combined model. However, from the perspective of slip instability, the slip phenomenon occurs on all the upper contact surface of the four models, and no through-slip occurs on the lower contact surface. These results indicate that the change in rock parking thickness has little effect on slip instability.

**Fig 15:**
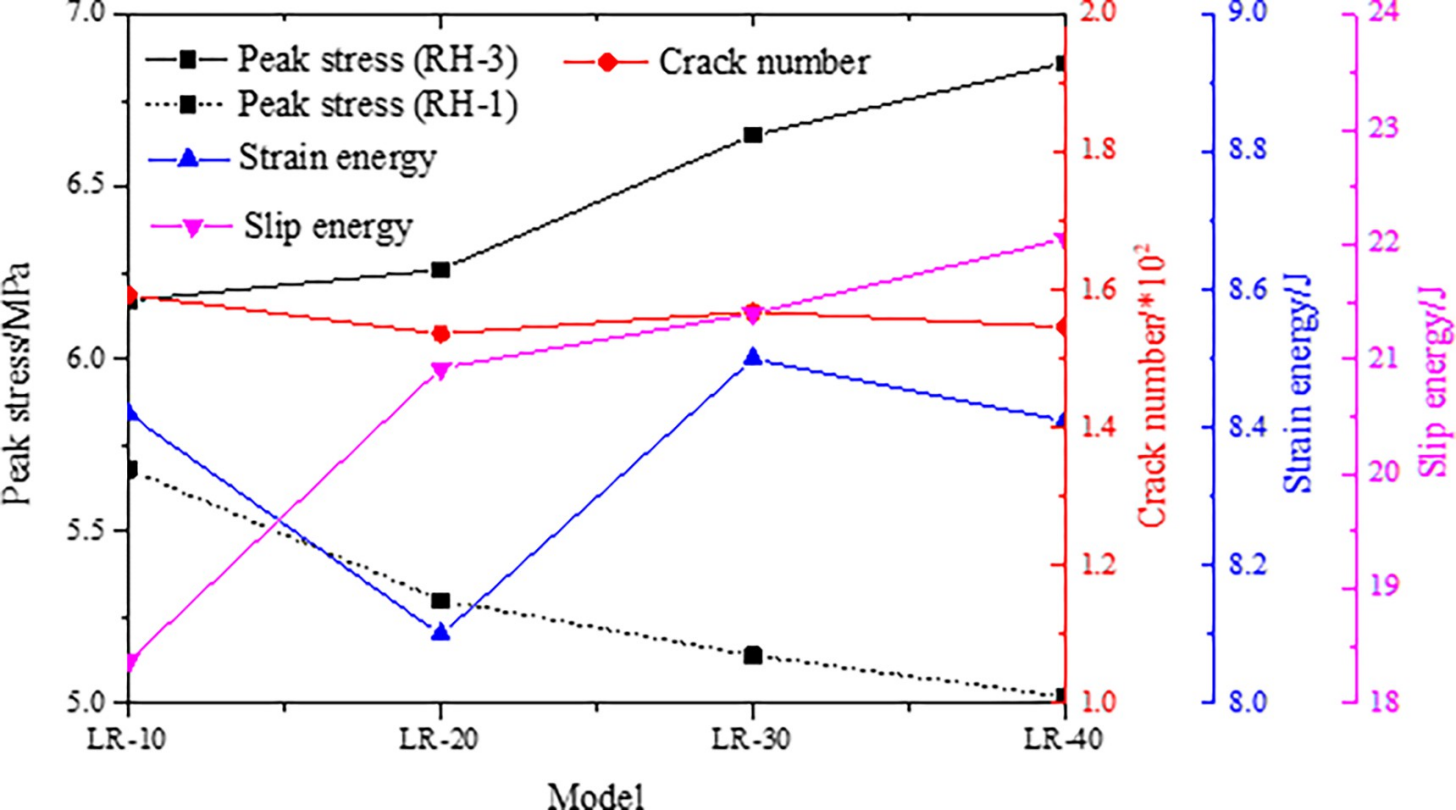
Parameter evolution characteristics of models with different thickness of rock parting.

The peak stress, number of cracks, strain energy and slip dispersion in the failure process of the combined models with different rock parting thickness are monitored, and the results are shown in Fig 15.

The instability strength of the models gradually increases with increasing thickness of the rock parting, and the number of cracks basically remains unchanged. This indicates that an increase in the thickness of the rock parting can enhance the stability of and increase the amount of elastic energy accumulated in the model. When the whole model is unstable, the elastic energy accumulated is released rapidly, which results in an increase in slip dissipation energy and destructive power. It is worth noting that the strength of the RH-3 model is greater than that of coal, and the peak strength of the model after instability increases with increasing coal thickness. When the strength of the model is lower than that of coal (model RH-1), the peak strength after instability of the model decreases with increasing coal thickness, and the rockburst risk gradually decreases. Therefore, parameters such as the thickness and strength of the rock parting should be detected in advance at the project site. Rockburst disasters are more likely to occur in area with higher strength and thicker rock parting.

## 5 Conclusion

1. Compared with the single coal structure or the coal-rock combined structure, the CRCS directly reflects the geological structure characteristics, and its instability precursor characteristics and influencing factors can better reveal the failure and instability process, which can provide a reference for the study of the rockburst mechanism in the bifurcation area of coal seam.
2. Slip can promote a change in fracture growth from an integral through fracture to a vertical fracture along the contact surface. The main failure form of fracture is tensile failure, but the dissipated energy is mainly released through shear failure. Slip can reduce the peak strength of the CRCS, and the model instability is characterized by low strength and high energy release, which increases the difficulty of predicting the rockburst of structural instability in the bifurcation area.
3. Before the instability of the CRCS, there are several precursor signal characteristics, such as the shortened development time of the "stable—fracture—stable" cycle, the abnormal slip dislocation of the contact surface, and the rapid accumulation of rock fracture energy. These characteristics can be used to predict rockburst accidents caused by the instability of coal seam structures in bifurcated areas.
4. The instability characteristics of CRCS are affected by the inclination angle of the contact surface, the strength and thickness of the rock parting. The inclination angle of the contact surface affects the instability form, the strength of the rock parting affects the instability state, and the thickness of the rock parting affects the impact tendency. Therefore, for coal seam in the bifurcated area with large contact angle, high strength and thickness, special attention should be given to preventing the occurrence of rockburst with structural instability.

## References

[1] Wojtecki Q, Konicek P, Schreiber J. Effects of torpedo blasting on rockburst prevention during deep coal seam mining in the Upper Silesian Coal Basin. Journal of Rock Mechanics and Geotechnical Engineering. 2017;9(4):694–701.

[2] Yardimci A G, Karakus M. A new protective destressing technique in underground hard coal mining. International Journal of Rock Mechanics and Mining Sciences. 2020;130:104327.

[3] Liu G J, Mu Z L, Karakus M. Coal burst induced by rock wedge parting slip: a case study in Zhaolou coal mine. International Journal of Mining Reclamation & Environment. 2018;32(5):297–311.

[4] Lu C P, Liu G J, Liu Y, Zhang H. Mechanisms of Rockburst Triggered by Slip and Fracture of Coal–Parting–Coal Structure Discontinuities. Rock Mechanics and Rock Engineering. 2019;52(9):3279–3292.

[5] Frid V. Calculation of electromagnetic radiation criterion for rockburst hazard forecast in coal mines. Pure and Applied Geophysics. 2001;158(5–6):931–944.

[6] Wojtecki L, Iwaszenko S, Apel D B, Cichy T. An Attempt to Use Machine Learning Algorithms to Estimate the Rockburst Hazard in Underground Excavations of Hard Coal Mine. Energies. 2021;14(21):6928.

[7] Fan Y F, Xiao X C, Xu J, Ding X, Wang A W, Wang B F, et al. Failure characteristics and conditions of rock-coal combination structure with weak layer under dynamic and static stresses. Scientific Reports. 2023;13(1):12410. doi: 10.1038/s41598-023-39427-5 37524999 PMC10390601

[8] Gong F Q, Ye H, Luo Y. The Effect of High Loading Rate on the Behavior and Mechanical Properties of Coal-Rock Combined Body. Shock and Vibration. 2018;4374530,.

[9] Wang T, Ma Z G. Research on strain softening constitutive model of coal-rock combined body with damage threshold. International Journal of Damage Mechanics. 2021; 31(1):22–42.

[10] Jiang Y D, Wang T, Song Y M, Wang X, Zhang W. Experimental study on the stick-slip process of coal-rock composite samples. Journal of China Coal Society. 2013;38(02):177–182.

[11] Willams T J, Wideman C J, Scott D F. Case-history of a slip-type rockburst. Pure and Applied Geophysics. 1992; 139(3–4):627–637.

[12] Chen S J, Yin D W, Jiang N, Wang F, Zhao Z H. Mechanical properties of oil shale coal composite samples. International Journal of Rock Mechanics and Mining Sciences. 2019; 123:104120.

[13] Song H Q, Zuo J P, Liu H Y, Zuo S H. The strength characteristics and progressive failure mechanism of soft rock-coal combination samples with consideration given to interface effects. International Journal of Rock Mechanics and Mining Sciences. 2021;138:104593.

[14] Liang B, Wang D, Jiang Y J, Sun X M, Luan H J, Wang C S, et al. Evaluating Fractal Damage and Acoustic Emissions of Soft Rock–Coal Combinations in a Deep Mining Area. Processes. 2023;11(9):2599.

[15] Tan Y L, Liu X S, Shen B, Ning J G, Gu Q H. New approaches to testing and evaluating the impact capability of coal seam with hard roof and/or floor in coal mines. Geomechanics and Engineering. 2018;14(4):367–376.

[16] Ma Q, Tan Y L, Liu X S, Gu Q H, Li X B. Effect of coal thicknesses on energy evolution characteristics of roof rock-coal-floor rock sandwich composite structure and its damage constitutive model. Composites Part B: Engineering. 2020;198:108086.

[17] Li F X, Yin D W, Wang F, Jiang N, Li X L. Effects of combination mode on mechanical properties of bimaterial samples consisting of rock and coal. Journal of Materials Research and Technology. 2022; 19:2156–2170.

[18] Chai Y J, Dou L M, Cai W, Małkowski P, Li X W, Gong S Y, et al. Experimental investigation into damage and failure process of coal-rock composite structure with different roof lithologies under mining-induced stress loading. International Journal of Rock Mechanics and Mining Sciences. 2023;170:105479.

[19] Alzo’ubi A K. The effect of tensile strength on the stability of rock slopes. Ph.D. thesis, University of Alberta, Edmonton, Canada, 2009.

[20] Gao F Q, Stead D, Kang H P. Simulation of roof shear failure in coal mine roadways using an innovative UDEC Trigon approach. Computers and Geotechnics. 2014;61:33–41.

[21] Naji A M, Rehman H, Emad M Z, Yoo H. Impact of Shear Zone on Rockburst in the Deep Neelum-Jehlum Hydropower Tunnel: A Numerical Modeling Approach. Energies. 2018;11(8):1935.

[22] Liu G J, Zhang H, Zhu Y W, Cao W H, Ji X J, Lu C P, et al. Investigations of Coal-Rock Parting-Coal Structure (CRCS) Slip and Instability by Excavation. Shock and Vibration. 2021; 1715644.

[23] Vardar O, Wei C C, Zhang C G, Canbulat I. Numerical investigation of impacts of geological faults on coal burst proneness during roadway excavation. Bulletin of Engineering Geology and the Environment. 2022;81(1):2.

[24] Liu W R, Yuan W, Yan Y T, Wang X. Analysis of Acoustic Emission Characteristics and Damage Constitutive Model of Coal-Rock Combined Body Based on Particle Flow Code. Symmetry-Basel. 2019;11(8):1040.

[25] Cao J S, Dai Q, Zhou Y, Ma D. Failure mechanism and strength of coal-rock combination bodies considering dip angles and fractal characteristics of interface. Journal of Central South University (Science and Technology). 2018;49(01):175–182.

[26] Nicksiar M, Martin C D. Factors Affecting Crack Initiation in Low Porosity Crystalline Rocks. Rock Mechanics and Rock Engineering. 2014;47(4):1165–1181.

[27] He Z L, Lu C P, Zhang X F, Guo Y, Meng Z H, Xia L. Numerical and Field Investigations of Rockburst Mechanisms Triggered by Thick-Hard Roof Fracturing. Rock Mechanics and Rock Engineering. 2022;55(11): 6863–6886.

[28] Itasca. UDEC User’s Manual [CP]. Itasca consulting group, Inc. 2014.

[29] Zhang H, Zhang Y G, Liu G J, Zhu YW, Ji X J, Cao W. H. Numerical study on the characteristics of roadway failure and instability in coal seam with rock parting. Scientific Reports. 2024;14(1):1587. 38238387 10.1038/s41598-024-51270-w PMC10796363

[30] Hoek E, Martin C D. Fracture initiation and propagation in intact rock-a review. Journal of Rock Mechanics and Geotechnical Engineering. 2014;6(4):287–300.

[31] Zhang H, Lu C P, Liu B, Liu Y, Wang H Y. Numerical investigation on crack development and energy evolution of stressed coal-rock combination. International Journal of Rock Mechanics and Mining Sciences. 2020;133:1365–1609.

[32] Yu Y J, Liu J J, Yang Y T, Wang P B, Wang Z M, Song Z Y, et al. Failure energy evolution of coal–rock combination with different inclinations. Scientific Reports. 2022;12(1):19455. doi: 10.1038/s41598-022-23813-6 36376443 PMC9663588

[33] Wang K, Fu Q, Xu C, Ai Z B, Li D, Shu L Y. The strength characteristics and competitive failure mechanism of primary coal-rock combination considering interface damage quantity. Fuel. 2023;352:129057.

[34] Xia Z G, Liu S, Zhuang B, Song J H, Fan F, Jiang N. Mechanical Properties and Damage Characteristics of Coal-Rock Combination with Different Dip Angles. KSCE Journal of Civil Engineering. 2021;25(5):1687–1699.

[35] Cheng Z B, Li L H, Zhang Y N. Laboratory investigation of the mechanical properties of coal-rock combined body. Bulletin of Engineering Geology and the Environment. 2020;79(4):1947–1958.

